# A new climate for genomic and epigenomic innovation in grapevine

**DOI:** 10.1186/s43897-025-00171-1

**Published:** 2025-05-12

**Authors:** Maximilian Schmidt, Timo Strack, Haylie Andrews, Lee T. Hickey, Peter A. Crisp, Kai P. Voss-Fels

**Affiliations:** 1 Department of Plant Breeding, Hochschule Geisenheim University, Geisenheim, Germany; 2 School of Agriculture and Food Sustainability, Faculty of Science, The University of Queensland, St Lucia, Australia; 3 Centre for Crop Science, Queensland Alliance for Agriculture and Food Innovation, The University of Queensland, St Lucia, Australia

## From challenge to action: grapevine improvement in the face of climate change

For thousands of years, wine has captivated cultures worldwide. Yet today, it is facing an existential threat as climate change casts a shadow over future production. Despite its remarkable plasticity, grapevine (*Vitis vinifera* L.) production is being pushed to its limits in many wine-growing regions worldwide due to more frequent extreme weather events such as heatwaves, floods, droughts, frosts, and hail (IPCC, 2021). These adverse conditions have led to reduced yields and compromised fruit quality. Globally significant cultivars are nearing the limits of their optimal growth conditions in traditional regions, threatening their valued wine characteristics and raising serious concerns about the future of the wine industry (Jones et al., 2012).

While regions near the 50 th latitude may temporarily benefit from warmer climates, long-term suitability is also at risk, albeit at a slower pace compared to Mediterranean regions (van Leeuwen et al. 2024). Biotic stressors, such as expanding populations of tissue-feeding and virus-transmitting insects, as well as higher fungal disease pressures, compound these challenges. Social and political pressures to reduce pesticide use further complicate the scenario.

The future of the grapevine industry hinges on leveraging multi-omic technologies and predictive breeding tools to accelerate genetic improvements for commercially valuable traits. Strategies include improving existing popular varieties through modern molecular technologies or developing new crossbred cultivars that are resilient to biotic and abiotic stresses. However, the wine industry and consumers remain hesitant about precision molecular breeding techniques and new released cultivars, despite improved disease tolerance.This commentary underscores the potential and necessity of adopting genomics, epigenomics, and new genomic technologies (NGTs) for accelerated grapevine improvement to secure the future of viticulture.

Adaptation to changing growing conditions has long been a hallmark of viticulture. Most commercially important cultivars are the result of hundreds to thousands of years of vegetative propagation coupled with directional selection for desired agronomic and oenological characteristics. Since the twentieth century, North American *Vitis* species have been crossed with European varieties to introduce resistance genes for powdery (*Erysiphe necator*) and downy (*Plasmopara viticola*) mildews and to control phylloxera (*Daktulosphaira vitifoliae*)—the latter achieved by grafting fruit-bearing scion cultivars with phylloxera-resistant rootstock varieties. Today, breeding programs focus on pyramiding resistance genes, a practice common in other perennial and annual crops, while simultaneously improving agronomic and oenological traits. However, climate change introduces additional complexities, including new priorities and breeding goals such as enhancing water-use efficiency for drought resilience, improving root and cluster morphology to optimize resource uptake and developing resistance to new pests and diseases.

## Improving traditional cultivars via clonal selection

**Fig. 1:**
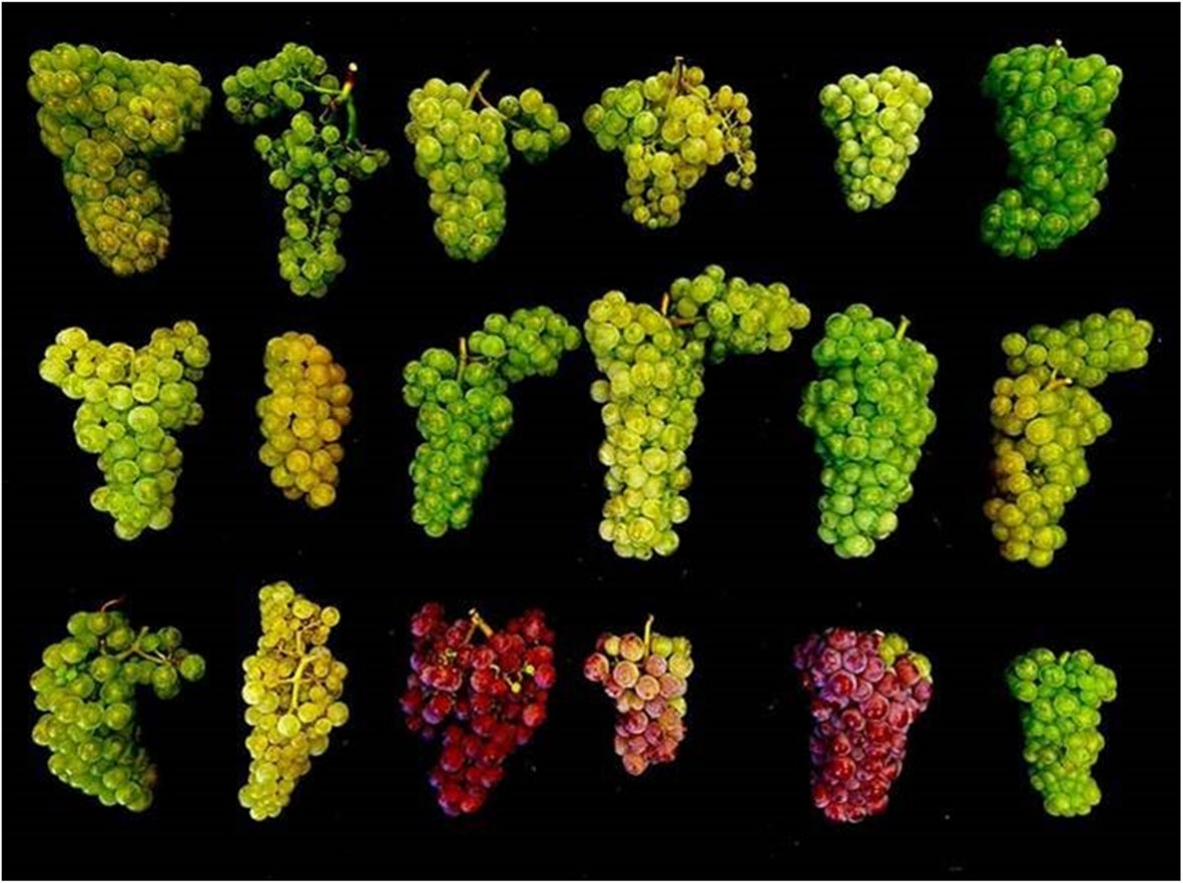
Phenotypic variation for bunch traits in 18 clones of the cultivar Riesling. Bunches were sampled in the field station at Hochschule Geisenheim University on 06/09/2024. Clones were collected from old vineyards over the last 30 years across different locations in Germany, France and Luxembourg. The standard set of nine simple sequence repeats markers (VVS2, VVMD5, VVMD7, VVMD25, VVMD27, VVMD28, VVMD32, VrZAG62 and VrZAG79) that is commonly used for cultivar recognition (Maul and Töpfer, 2015) identified all clones as Riesling. The colour mutant Red Riesling cannot be differentiated from White Riesling using those markers

Centuries of vegetative propagation of grapevine cultivars (e.g. 600 years in White Riesling and over 1,200 years in Pinot noir) led to an accumulation of molecular variation that has translated into substantial amounts of variation for a range of mono-, oligo- and polygenetic commercially relevant traits. For example, broad diversity for bunch traits like architecture, size and colour can be found in the cultivar Riesling (Fig. 1).

Initially, grapevine breeding used clonal selection as a means to overcome yield reduction due to virus infections by selecting healthy, virus-free planting material. Subsequently, this practice has then been used to also systematically exploit complex trait variation to identify, propagate and commercialise new clonal variants of traditional cultivars with improved trait characteristics. As a result, there are dozens to hundreds of commercially available clones for virtually all important grapevine cultivars. Although it is assumed that genetic mutations and likely epigenetic variation are the causal drivers, the molecular mechanisms that shape complex trait variation within traditional cultivars are vastly unknown.

From today’s perspective, this makes the whole practice of clonal selection very slow and inefficient, mainly because it relies on i) chance that favourable and stable molecular variants have arisen and ii) that they can be identified and selected effectively. Like many crops, mutagenesis has been adopted in grapevine improvement as a means to induce novel variation, although the random nature of non-targeted mutations makes it challenging to develop new clones with desirable trait configurations.

## Cross-breeding new fungus-resistant cultivars

Developing new varieties via targeted hybridization can greatly enhance the range of trait diversity outside those available through clonal variation within single cultivars of *Vitis vinifera*. However, this requires the adoption of new varieties that are assumed to lack traditional fruit characteristics and regional wine identities, which are presumed to be potentially disruptive to the industry. The effort to develop mildew-resistant grapevine varieties began with the introduction of resistance genes from North American and Asian *Vitis* species into *V. vinifera*. This process was difficult and slow, hindered by long breeding cycles, inbreeding depression, and by traits inherited from wild *Vitis* species that caused lower wine quality. Early resistant cultivars, such as the French hybrids, faced strong opposition due to their distinctive “hybrid taste” which compromised their acceptance among growers and consumers, leading to a political ban in many wine-growing nations.

Over time, advancements in breeding techniques allowed for rigorous selection for desired flavours, ensuring that resistance could be achieved without compromising wine quality. Today, global breeding programs have produced new generations of cultivars that combine high-quality wine profiles with disease resistance and that could therefore significantly improve viticulture's ecological and economical sustainability. Several factors are now assumed to drive the need for adopting new, resilient grapevine varieties. These include a strong need to improve sustainability in viticulture, particularly due to the phase-out of key fungicides, emerging fungicide-resistant pathogens, and societal demands for reduced chemical use in agriculture aligning with policies like the European Green Deal, which mandates a 50% reduction in pesticide use by 2030.

Furthermore, rising temperatures that cause altered wine styles, along with an increasing frequency of extreme weather events like droughts, heatwaves, and downy mildew epidemics pose significant risks to grape production. While the cultivation of traditional varieties remains attractive due to market familiarity, transitioning to disease-resistant cultivars is critical for ensuring the long-term resilience and sustainability of the wine sector (Töpfer and Trapp 2022). Continued breeding innovations, however, are needed to accelerate the slow breeding progress of typically at least 25 years until a new improved cultivar can be released to the market.

## Barriers to the adoption of new breeding technologies

New tools that that are gaining traction in other agricultural species offer opportunities to accelerate the grapevine improvement process, for example, new genomics technologies, new high-throughput phenotyping tools and predictive breeding approaches like genomic selection. Integrating these techniques can significantly help to uncover the genetic basis of desirable traits and accelerate the breeding process. This also forms the technological basis to identify molecular variants that gene editing can target, a concept particularly appealing for improving traditional varieties.

However, resistance to new technologies in viticulture stems from the cultural and historical significance of traditional varieties, public scepticism of genetically modified organisms, as well as regulatory restrictions on the evaluation and production of GMO crops. New genomic techniques (NGT) like CRISPR-Cas9 and related technology variants are highly promising methods to preserve traditional varieties by making precise genomic modifications to specifically alter agronomic or resistance traits while retaining their varietal identity. However, the genetic basis of many commercially relevant traits remains vastly unknown in grapevine and further research is needed to facilitate the discovery of potential genome editing targets.

Furthermore, the choice of the breeding tool should always be guided by the trait that the breeding program is targeting, rather than the reverse. For instance, improvements through genome editing are likely more attainable for simple traits rather than complex traits. With the right legal framework, results from NGTs could be considered new clones of traditional cultivars, ensuring the preservation of both quality and heritage in the face of climate change. Adoption of new NGTs however will largely depend on legislative concessions that allow edited clones to retain their status as renowned varieties. The preservation of distinguishable traits and world-renowned varieties is of the utmost importance to growers and must be considered when integrating new technologies.

## Inter- and intra-varietal variation forms the basis for future adaptation

**Fig. 2:**
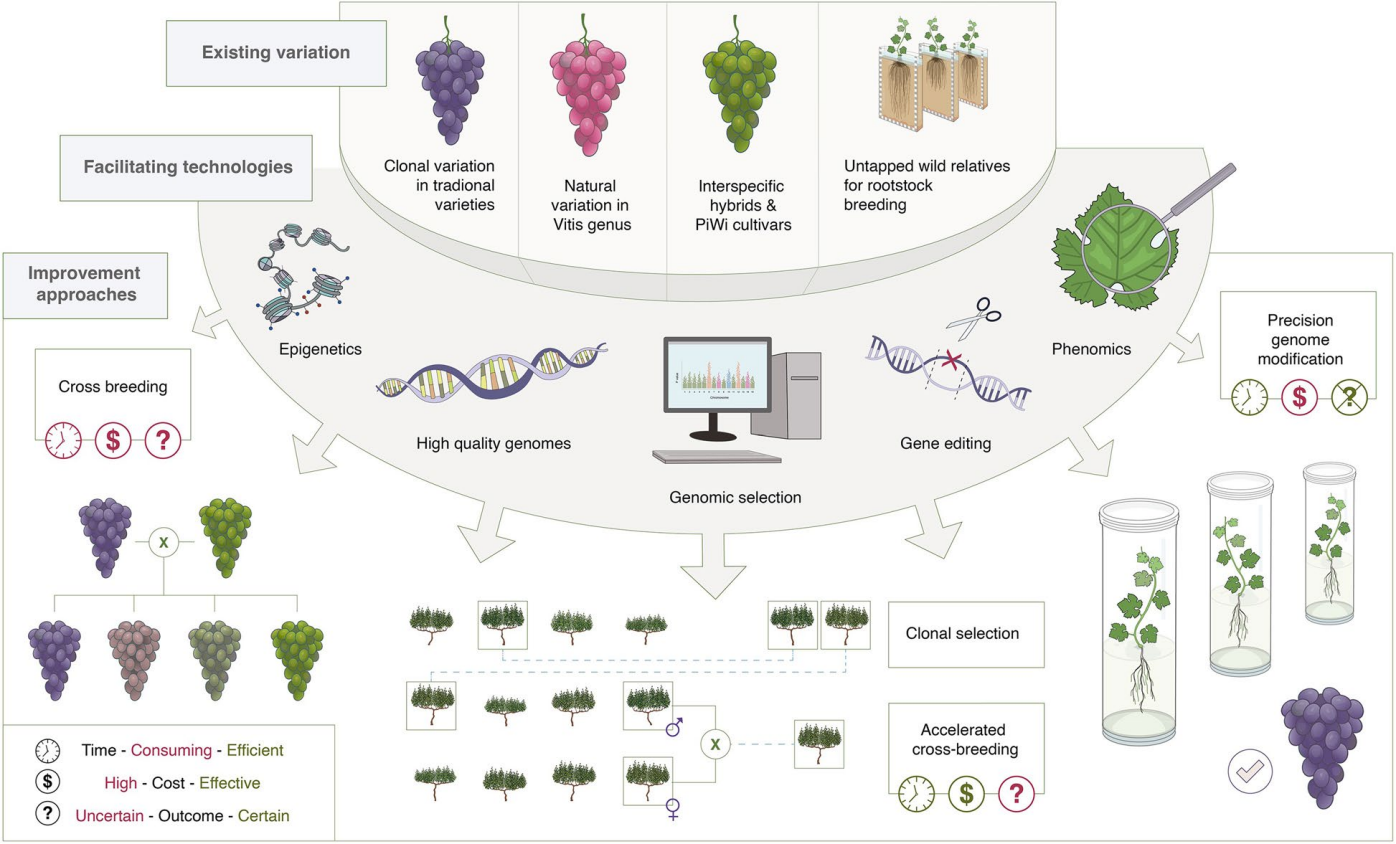
Overview of the main grapevine breeding strategies and the improvement potential of modern breeding tools. Traditional grapevine breeding uses clonal selection and crossbreeding for scion and rootstock improvement, where conventional breeding cycles take at least 25 years. Efficiency could be greatly improved through the successful integration of new technologies including genomics, epigenomics, biotechnology and predictive breeding

The key sources of variation for grapevine breeding include natural variation within the *Vitis* genus, clonal variation within traditional varieties (Fig. 1), and new artificially induced variation via classical mutation breeding or targeted modifications using NGTs. Traditional methods for improving grapevine, such as cross-breeding new varieties or developing improved varietal clones, are extremely time-intensive, costly and sometimes inefficient. With conventional methods and breeding cycles lasting over 25 years, there are rising concerns about the ability to tackle climate change, shifting demographics, societal demands and changing regulations. We propose that modern predictive breeding tools and NGTs informed by multi-omics data offer new avenues for innovation, as summarised in Fig. 2.

The challenge lies in accelerating the discovery and incorporation of useful genetic variation into breeding, which requires coordinated national and global collaborations. Despite the rich genetic diversity within and between accessions, new approaches are needed to accelerate the genetic improvement process. Recent genomic technologies, including high-quality genome sequencing, genomic selection, and methylation pattern identification, could enable rapid and early identification of breeding material with high genetic merit. When combined with phenotyping tools, these methods can also facilitate targeted genome editing (Fig. 2).

## New genomic and epigenomic technologies open the way for accelerated grapevine improvement

Over the past five years, a growing number of reference genomes for widely grown *V. vinifera* varieties including Shiraz (Onetto et al. 2023) and Malbec (Calderon et al. 2024) have been published. These complement the grapevine reference genome, originally thought to be based on a self-pollinated clone of Pinot noir, but later revealed to be misidentified as the result of cross-pollination with the variety ‘Helfensteiner’ (The French–Italian Public Consortium for Grapevine Genome Characterization 2007), which has been continuously updated to the current telomer-to-telomer version (Shi et al. 2023). For this genome assembly, high-quality gene models are available with many of these being manually curated based on Illumina and PacBio Iso-seq data thanks to the efforts of the grapevine research community.

Moreover, the development of pangenomes over stand-alone reference genomes allows for the identification of agronomically relevant structural variants not previously catalogued, which when combined with other genomic indicators of functionality can narrow the scope for sequence targets for either breeding or precision engineering (Liu et al. 2024). These genomes and gene models serve as references for large-scale genotyping of traditional grapevine varieties, revealing intra-varietal genomic variation. When combined with modern phenotyping technologies, this approach can uncover the genetic basis of traits such as loose-cluster architecture, drought tolerance, and delayed berry ripening. This insight can aid in accelerating variety development via genomics-assisted selection, or in the use of NGTs for designing improved clones of traditional varieties while preserving their distinct characteristics under the right legal framework (Fig. 2).

Genomic technologies coupled with quantitative genetics approaches can help identify the molecular determinants of intra-varietal variation, including the roles of genetic and epigenetic mechanisms. Additionally, the growing accessibility of long-read sequencing accelerates breeding and uncovers genomic phenomena previously difficult or impossible to detect, such as haplotype phasing or specific cell layer contributions. This opens new ways to study genetic and epigenetic architectures of traits as a basis for new breeding strategies and has the potential to greatly improve the current challenges in grapevine genetic improvement.

Indeed, another blind spot for grapevine improvement so far is the influence of epigenetic modifications on phenotypic traits. Work in many species has demonstrated extensive natural variation, including variation impacting agronomic traits in grapevine (Dal Santo et al. 2018). This variation, such as that documented in DNA methylation, can be associated with phenotype differences and can capture alleles missed by sequence only analyses (Xu et al. 2019). DNA methylation also plays a well-known role in fruit ripening (Zhang and Zhu, 2025), with evidence for a role in grapevine also (Shangguan et al. 2020). These prior investigations all successfully used the classic approach of bisulfite or EM-sequencing coupled to short-read technologies. Other investigations in grapevine have also used variations of reduced representation methylation profiling to investigate the epigenome (Tan and Rodríguez López 2023), although these are limited by the small fraction of the genome profiled.

Recent sequencing technologies, such as Oxford Nanopore and Pacific Biosciences, have simplified and enhanced this process by simultaneously generating epigenetic and genomic information. By analysing native DNA modifications, these methods eliminate the need for additional experiments, enabling the direct assessment of genetic variants and methylation status in specific genomic regions. Besides providing obvious benefits in understanding epigenetic causes for phenotypic variation, this also provides insights into understanding epigenetic adaptation to different environments. There is already evidence that epigenetic modifications play a role in adapting grapevines to different environments by regulating the expression of genes involved in responding to environmental stimuli, as an Australian study about the epigenetic differences in Shiraz vines planted in different vineyards shows (Xie et al. 2017). Novel variation could also be induced, for example, using DNA methylation inhibitors or targeted modifications to produce epialleles which control the expression of genes of interest.

Latest-generation genomics and epi-genomics tools also form the basis for identifying targets for genomic editing approaches which show enormous potential for grapevine improvement, particularly with regards to developing new variety clones via targeted alteration of agronomic or resistances traits without negatively impacting oenological quality (Fig. 2). Modern NGTs have been highly limited in grapevine due to a lack of knowledge of trait genetic architectures and gene functions, technical difficulties and an aversion of the industry and market to the technology. However, recent research demonstrates impressive potential, for example, in a recent study that describes the improvement of downy mildew resistance in Sugraone and Crimson seedless via genome editing (Giacomelli et al. 2023). Similar progress improving downy mildew resistance in different rootstock varieties and Syrah has been made using NGTs (Djennane et al. 2023).

Because of grapevine’s amenability to grafting, there are also exciting possibilities to adapt recent advances in de novo meristem induction (Maher et al.^ 2019^) to generate gene edited branches or to incorporate graft transmissible gene editing via donor rootstocks (Yang et al. 2023). One could imagine developing custom-designed rootstocks with different genome-edited traits, such that gene editing could be undertaken merely by grafting the desired scion onto the engineered root. Epigenome editing for heritable trait improvement in the absence of DNA sequence differences is also a possibility, as recently demonstrated for disease resistance in another clonally propagated species, cassava (Veley et al. 2023).

Finally, cost-efficient large-scale profiling of genetic and epigenetic variation in breeding populations can become highly useful as a basis to train prediction models for complex traits, which in turn can enable more accurate and faster selection decisions in grapevine improvement, thereby substantially shortcutting the extremely slow conventional breeding process. The modern breeding tool genomic selection has revolutionised breeding of crops and livestock and delivered increases in genetic gains that could not be achieved with conventional selection strategies (Hickey et al. 2017). Few studies have adopted the principle in grapevine yet, though the first results are highly promising and show that commercially relevant traits can be predicted at considerably high accuracies (Brault et al. 2024). For grapevine, as for other perennial species with extremely long generation and breeding cycle times, predictive breeding methods like genomic selection could deliver substantial genetic gains compared to conventional breeding approaches that purely rely on phenotypic assessments of target traits. High-throughput phenotyping approaches, such as multi- or hyperspectral and aerial imaging, show promise for accelerating predictive breeding in grapevine, especially when combined with genomic data (Magon et al., 2023, Brault et al. 2024).

Using the latest long-read sequencing technologies, both genetic and epigenetic variants can now be called simultaneously. Coupled with genetic imputation approaches to design high-throughput genotyping pipelines, this could be used for large-scale profiling of genetic and epigenetic variation in grapevine populations, both within and across cultivars, as demonstrated in dairy cattle (González-Recio et al. 2023). Although there is no published work on this in grapevine yet, pioneering studies imply that epigenetics must play an important role in complex trait variation (e.g. comprehensively reviewed in Berger et al. 2023), and hence could provide improved predictive ability in genomic selection. The development of genomic and epigenomic prediction frameworks could therefore greatly facilitate the selection of improved clones of traditional cultivars as well as the design of new cross-bred cultivars that combine resilience and oenological potential.

## From tradition to transformation: sustaining viticulture through modern breeding technologies

Grapevine is one of the world’s oldest and most iconic crops, deeply embedded in the cultural and historical heritage of many wine-growing regions. However, the sustainability of grapevine production is increasingly threatened by climate change, which exacerbates challenges to yield stability, grape quality, and regional production systems. Concurrently, there is growing pressure to reduce the environmental footprint of viticulture, as both conventional and organic farming systems remain heavily reliant on intensive plant protection inputs.

Recent advances in genetics and genomics offer significant opportunities to adapt grapevine to these challenges through precision molecular approaches and predictive breeding frameworks. Targeted hybridization has shown promise for developing new cultivars with substantially improved trait configurations, such as stress resilience and disease resistance. Although the adoption of new varieties has faced resistance due to deviations from traditional characteristics, established regional identities and key elements of the wine market, current efforts are addressing this challenge by balancing innovation with the preservation of these defining traits. Further solutions include ongoing efforts to study the wine characteristics of new cultivars and optimize their production to ensure alignment with established quality standards and regional identities.

A complementary approach involves the precise modification of traditional cultivars to introduce novel, favourable traits while preserving their defining varietal characteristics. Gene-editing technologies, in particular, provide a means for targeted trait improvement without altering the genetic identity that underpins varietal branding. Despite this potential, identifying specific genes and molecular targets for editing remains a substantial challenge. Advanced genomic and epigenomic tools are critical to addressing this gap, enabling the dissection of complex traits and facilitating trait-prediction models that can drive future breeding innovations.

To fully capitalize on these opportunities, increased investment in research and innovation is essential. Long-term funding is required to expand foundational knowledge, translate molecular tools into breeding programs, and support field validation of new cultivars or edited varieties. Additionally, economic and socio-political hurdles such as regulatory restrictions, public acceptance, and market dynamics must be addressed to enable the implementation of modern breeding technologies at scale.

Grapevine exemplifies a broader need across other important perennial species to integrate cutting-edge genetics and genomics technologies into breeding efforts. By overcoming these scientific, economic, and policy-related challenges, the viticulture industry can better adapt to climate change, reduce its environmental impact, and ensure the continued economic and cultural viability of grapevine production systems.

## Data Availability

Data sharing is not applicable to this article as no datasets were generated or analysed during the current study.
